# Evolving academic and research partnerships in global health: a capacity-building partnership to assess primary healthcare in the Philippines

**DOI:** 10.1080/16549716.2023.2216069

**Published:** 2023-05-30

**Authors:** Anu Aryal, Fernando B. Garcia, A. J. Scheitler, Emerito Jose A. Faraon, T. J. Robinson T. Moncatar, Ofelia P. Saniel, Fely Marilyn E. Lorenzo, Roberto Antonio F. Rosadia, Riti Shimkhada, James Macinko, Ninez A. Ponce

**Affiliations:** a Center for Health Policy and Research, University of California Los Angeles, Los Angeles, CA, USA; b Department of Health Policy and Management, Fielding School of Public Health, University of California Los Angeles, Los Angeles, CA, USA; c Department of Health Policy and Administration, College of Public Health, University of the Philippines Manila, Manila, Philippines; d Department of Epidemiology and Biostatistics, College of Public Health, University of the Philippines Manila, Manila, Philippines

**Keywords:** Capacity building, research collaboration, primary care, Philippines, non-communicable diseases

## Abstract

Building fair, equitable, and beneficial partnerships between institutions collaborating in research in low- and middle-income countries (LMIC) and high-income countries (HIC) has become an integral part of research capacity building in global health in recent years. In this paper, we offer an example of an academic collaboration between the University of California Los Angeles, Center for Health Policy and Research (UCLA CHPR) and the University of Philippines, Manila, College of Public Health (UPM CPH) that sought to build an equitable partnership between research institutions. The partnership was built on a project to build capacity for research and produce data for policy action for the prevention and care of non-communicable diseases (NCDs) through primary healthcare in the Philippines. The specific objectives of the project were to: (1) locally adapt the Primary Care Assessment Tool for the Philippines and use the adapted tool to measure facility-level primary care delivery, (2) conduct focus group discussions (FGDs) to gather qualitative observations regarding primary care readiness and capacity, and (3) conduct a comprehensive population-based health survey among adults on NCDs and prior healthcare experience. We describe here the progression of the partnership between these institutions to carry out the project and the elements that helped build a stronger connection between the institutions, such as mutual goal setting, cultural bridging, collaborative teams, and capacity building. This example, which can be used as a model depicting new directionality and opportunities for LMIC-HIC academic partnerships, was written based on the review of shared project documents, including study protocols, and written and oral communications with the project team members, including the primary investigators. The innovation of this partnership includes: LMIC-initiated project need identification, LMIC-based funding allocation, a capacity-building role of the HIC institution, and the expansion of scope through jointly offered courses on global health.

## Introduction

While the ethics of academic partnerships in global health have been discussed for many years, there has been a more recent growing dialogue on different models of global health academic partnerships [1–5]. Initial global health partnerships, which focused primarily on sending medical trainees and student researchers from LMIC-HIC for short periods, are criticised for not being valuable to the local partners in LMIC [6]. The field has begun to shift its focus towards long-term partnerships beneficial to both parties [7–9].

Despite the shift, there are still numerous issues with the LMIC-HIC long-term academic partnership. One primary concern is the benefit-misalignment favouring HIC partners. Whether writing more publications, not having to face day-to-day field-level data collection and processes, or being remunerated at a higher rate, HIC partners get the upper hand in the partnership [2,10]. The partners in LMICs often feel engagement comes late in the process, mostly after things are in final shape, thus allowing little to no feedback and no opportunity to contextualise with the local setting [2,6,11]. Some have specifically called this the ‘colonial mindset,’ highlighting the notion that knowledge is power. The presumption of HIC academic institutions as holders of knowledge deemed ‘regimes of truth’ [12] creates the perspective that such institutions perpetuate power differentials in the partnership [1]. The academic community has called for greater engagement in research collaboration and authorship with LMIC partners including actions for decolonising global health [13]. One of the decolonising efforts would mean moving beyond thinking of the LMIC location as only a research site, the LMIC partner institutions only as research coordinators, and local researchers only as data collectors [14]. It requires meaningful engagement and sincere capacity-building efforts. Yet, a recent scoping review found that only three out of nine global health organisations mentioned capacity building/strengthening as a principle of their partnership [15].

It is an exciting era for new LMIC-HIC partnership programs to support research capacity in LMICs, especially with the availability of numerous grant opportunities focusing mainly on the capacity-building component. But for any institution venturing into this field, it is essential to consider building fair, equitable, and beneficial partnerships with the LMIC institution and people. In this paper, we have highlighted one such academic collaboration between the University of California Los Angeles, Center for Health Policy and Research (UCLA CHPR) and the University of Philippines Manila, College of Public Health (UPM CPH) that exemplifies the ‘decolonising global health’ practice in global health research. We adapted the conceptual model of Leffers and Mitchell to describe the partnership process [16]. This case study was written based on the review of project documents, including study protocols, and written and oral communications with the project team members, including the primary investigators. We do not claim this partnership case to be the best model, but rather as a valuable model that depicts new directionality and opportunities of LMIC and HIC academic partnerships.

## Conceptual model

**Figure 1.**
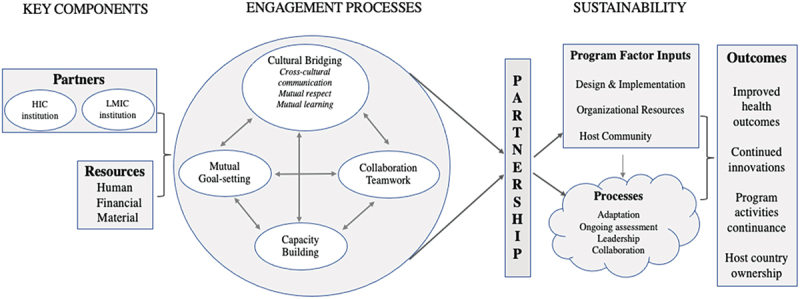
Conceptual model of the process of partnership in global health [16].

While Leffers and Mitchell (2011) developed a model for global health nursing partnerships, we find it equally applicable in our academic and research partnership described in this paper (see Figure 1). It has three premises: partnership components, engagement processes, and sustainability. In this case, a HIC institution and an LMIC institution find resources and enter into a partnership. The first step in the engagement process is understanding each other’s culture. Then a mutual goal is set, and the intervention is planned so that the tasks are completed collaboratively with each partner’s role clearly defined. A key focus is on the capacity building of the LMIC institution. To ensure the sustainability of the intervention, the program should consider local needs assessment in the design step and community participation. The intervention should be adapted in the local setting with regular evaluations. The LMIC institution should lead and own the program with collaboration from the HIC institution. The ultimate outcome of the partnership is improved health of the population. Additional outcomes such as continued innovation, program activities, and host country ownership are also crucial to the success of the partnership.

## The UCLA-CHPR and UPM-CPH partnership

The ‘Integrating Non-communicable Disease (NCD) Management in Primary Healthcare: A Population Health Survey and Action Initiative’ research capacity-building project between the University of California Los Angeles Center for Health Policy Research (UCLA CHPR) and the College of Public Health, University of Philippines Manila (UPM CPH) spanned over five years since 2018. This project was the foundation for establishing a formal UPM CPH and ULCA CHPR academic partnership. A key objective was to build research capacity of UPM CPH to build an empirical database and a system of data collection to assess the effectiveness of local health systems in the Philippines could be used by both health policymakers and the communities concerned.

## Partners and resources

Usually, in global academic partnerships, the universities in the HIC look for partners in LMIC to conduct research. However, this partnership formation was unique as an LMIC institution sought out the partnership. The UPM CPH, located in the capital city of the Philippines, is the pioneer and largest public health college in the nation and the oldest public health school in Asia. The leaders at the university envisioned developing a program and eventually transforming it to a Center for Health Equity within the college that will provide crucial evidence and give assistance to the government on health policy decision-making related to universal health coverage and primary healthcare. They determined that the UCLA CHPR’s research capacity, for example, its research processes, data collection, and data use practices, could be a learning ground for UPM CPH. The UCLA CHPR houses survey research expertise through its flagship California Health Interview Survey (CHIS), the largest population-based survey in any state of the US [17]. Working with UPM CPH was in line with UCLA CHPR’s mission and vision. UCLA CHPR’s stated vision is to democratise data and put into the hands of the public in ways that inform, educate, and result in policy change. The UCLA CHPR launched CHIS in 2001, which has been conducted on a regular basis since then and is made publicly available. Data from CHIS is used widely for policy making in the state. UCLA CHPR routinely works with organisations to support the development, improvement and sustainability of programs by creating high-quality health data informing evidence-based decision-making in policy development and program execution. A visit by the Chancellor of UPM to UCLA CHPR in 2018, reciprocated by the visit by the CHPR director and the Department of Health Policy chair from the UCLA School of Public Health, was the foundation of the partnership. A formal memorandum of understanding was signed in 2017.

Contrary to the global health funding landscape where the funding agencies are in HICs and often contribute to power asymmetry due to resources, this project identified funding opportunities in the LMIC. The Philippine California Advanced Research Institute (PCARI) is a project of the Philippine Government’s Commission on Higher Education (CHED). It provides funding to academic institutions in the Philippines and partner universities in the University of California (UC) system on collaborative projects aimed at increasing the research capacity of Philippine institutions. UCLA initiated a request to become a recognised UC institution for affiliation with PCARI to be eligible to apply for their joint funding opportunities. Once affiliation was granted in 2017, UPM and UCLA teamed up to apply for PCARI’s call for research proposals. This project, ‘Integrating Non-communicable Disease (NCD) Management in Primary Healthcare: A Population Health Survey and Action Initiative,’ was awarded funding by CHED-PCARI and commenced in October 2017. The award provided personnel, operating, and direct research implementation cost for UPM CPH and project-related personnel and operating cost for UCLA CHPR.

## Mutual goal setting and collaboration

While several surveys in the Philippines include valuable information on health conditions as well as healthcare expenditures, they are limited to population groups such as children and women, are conducted over a longer interval (3 to 5 years), or focus on specific health issues (nutrition or HIV). None of these existing surveys facilitated linkage to health facility-level data and primary care with accompanying information on access and use of healthcare. Based on the needs identified by the local stakeholders, the project aimed to build an empirical base of population health data coupled with health facility information that is relevant in assessing the effectiveness of local health systems in the Philippines. These data would identify key primary healthcare performance indicators to define levels of primary healthcare experience related to the prevention of NCD risk factors, for example, barriers to utilisation, costs associated with healthcare, perceived quality of care and NCD-specific care, and sociocultural and health-care related factors associated with the healthcare experience. This project was led and maintained by the Department of Health Policy and Administration in UPM CPH.

The overall objective of this project was to improve primary healthcare delivery in the prevention and care of non-communicable diseases (NCDs) in the Philippines on the local level. The specific objectives of the project were to: (1) locally adapt the Primary Care Assessment Tool [18] for the Philippines and use the adapted tool to measure facility-level primary care delivery at 330 facilities in 3 provinces, (2) conduct focus group discussions (FGDs) within three provinces and two cities to gather qualitative observations regarding primary care readiness and capacity, and (3) conduct a comprehensive population-based health survey among adults on NCDs and prior healthcare experience among approximately 4,600 households in two provinces. This project goes beyond assessing NCD-related conditions and hopes to contribute to national and local UHC implementation in full support of Republic Act 11,223 - An Act Instituting Universal Health Care for All Filipinos, Prescribing Reforms in the Health Care System, and Appropriating Funds Therefore (also known as the ‘Universal Health Care Act’) enacted in 2018. Peer-reviewed publications based on data collection study results from this project are currently underway and are being led by the investigators in the Philippines.

## The team and cultural bridging

Teams from UPM CPH and UCLA CHPR were formed with complementary expertise from both institutions. The UPM CPH team included one principal investigator, five co-investigators, and one research fellow. UPM CPH’s co-investigator team represented a diverse background with expertise in survey research, qualitative studies, policy analysis and research, and epidemiology/biostatistics. The UCLA CHPR team consisted of two primary investigators, an expert in survey design, an expert in primary healthcare, the director and assistant director of CHIS, a statistician and survey methodologist, a staff researcher, and a program manager. Moreover, UPM CPH built relationships with local researchers from partner universities – Tarlac State University, Cebu Normal University, and Western Mindanao State University to conduct local data collection, with the goal of building capacities in areas outside of Metro Manila and further build opportunities with them for policy development and capacity building.

It was important that the team members at UCLA CHPR understood the cultural context of the Philippines: both academic culture and community. One of the primary investigators from UCLA is of Filipino ancestry with multiple ties to the Filipino research community both in the US and the Philippines. Other team members had experience working in the Philippines or other LMICs. Similarly, at UPM CPH, the faculties had exposure to US academic culture, and in fact, two of the UPM CPH faculties had doctoral degrees from UC system universities. Additionally, the key team members from both sides visited each other’s site during the study, enhancing cultural familiarity and building rapport.

Project managers at each institution led the collaboration process. The managers were in regular communication regarding the status of the respective team’s tasks to ensure adherence to the research timeline and in collaborating on all reporting requirements. The teams at each institution participated in regular joint video conference calls, which occurred weekly to monthly depending on the level of consultation requested by the UPM team. The time for the call was set such that it was morning in Manila and afternoon in Los Angeles.

## Research implementation and capacity building

The proposal process was collaborative, with teams at UPM CPH and UCLA CHPR contributing to the overall concept development and writing process. The physical distance and the time difference were bridged by emails, file sharing for working documents, and video conferencing. This working arrangement continued as the proposed project’s design relied on expertise from the teams at each site sharing in all tasks. UCLA CHPR’s extensive experience in survey research was matched with the UPM CPH team’s expertise in the local population and health systems. The team planned to adapt the CHIS survey to the Philippine context by naming it the Philippine Public Health Survey, translating the survey into the relevant local languages spoken at the study sites, and implementing the survey as an in-person interview with an option of a web-based self-administered survey. The project was led, conducted, and managed by UPM CPH, while UCLA CHPR played a consulting role, providing content expertise on study design, questionnaire development, implementation of surveys, and other critical strategic tasks related to research development, study management, writing, and dissemination.

A critical objective of this partnership has been to strengthen the research capacity among public health researchers in the Philippines on health systems research, particularly around NCD service delivery. With the adaptation of the Primary Care Assessment Tool (PCAT) to the Philippines context and through the use of FGDs, the goal had been to identify gaps and areas of need in primary healthcare service to enhance primary care’s ability to address NCD prevention and control. The PCAT assessment and interviews determine primary healthcare readiness, particularly the capacity and ability of a local facility to deliver healthcare to the community [18]. This information helps local systems to launch better and targeted healthcare delivery changes to make quality health services more accessible. The planned household survey was designed to be a survey that could be done on a regular interval basis by local partner universities to collect population-based data for the country. We involved partner state universities in three provinces to be able to undertake surveys. The project oriented 13 faculty and trained 9 staff across the entire research cycle – from development to data collection to analysis to dissemination. These have included face-to-face as well as online-based workshops and seminars.

As mentioned above, the UCLA CHPR team’s involvement in the project was as a consultant, with a focus on building capacity for UPM CPH. UPM CPH was entirely responsible for data collection operations and had direct participant contact. Early activities by UCLA CHPR included helping with study design, including sample size calculations, drafting institutional review board application materials, questionnaire development, and program management guidance. As the project progressed, the UCLA CHPR team offered advice on adapting the study to challenges brought on by COVID-19, creating ‘terms of reference’ for working with private vendors for data collection, technology, and data storage guidance. Beyond technical improvements, capacity building requires community, funder and government engagement. Using the CHIS Participatory Planning model [19], UCLA CHPR encouraged UPM CPH to form an Advisory Group comprised national and local health managers, policy makers, academe, non-government organisations, and private sector health system thought leaders, supporting the unifying goal of strengthening the effectiveness of the Philippine health delivery systems. By the end of 2022, UPM CPH was successfully able to complete the survey from 152 health facilities in 3 provinces (Cebu, Tarlac, and Zamboanga del Sur), and conducted a total of 31 focus groups with a total of 113 participants from 72 health facilities from 2 provinces (Cebu and Tarlac).

## Joint-teaching activities

The ongoing research collaboration also created an opportunity to co-teach graduate academic courses offered to students at UPM CPH and UCLA Fielding School of Public Health (FSPH). Between 2018 and 2021, more than 20 graduate students in UCLA FSPH and more than 30 graduate students in UPM CPH were offered two classes (1) Global Health: Frameworks, Policy and Practice and (2) Health Economics: Low, Middle-Income Countries (LMIC) and High-Income Country (HIC) Perspectives. The Global Health course provided an overview of global health affairs systems and policies. Upon completion, students were expected to develop the competency to design capacity strengthening plans, develop strategies for forming collaborations and partnerships to address global health issues, identify a code of professional practice and ethics, understand matters of health equality and social justice principles in a variety of socio-cultural and political settings, and conduct strategic analysis on a variety of complex health systems. The Health Economics course focused on the application, caveats, limitations, and extensions of economic theories globally. The intent was to help UPM CPH and UCLA FSPH students benefit from instructors and classmates with realities and perspectives that differ from themselves, increase the experiences of global health students to interact, collaborate with, and learn from partners in diverse economic settings. To achieve this, the course had a synchronous structure, with classroom time accessed through an online video conference portal. Each class session had faculty members from each institution. Weekly discussion board assignments facilitated the dialogue of real-world and real-time challenges and potential solutions reflecting the assessments and perceptions of students in each country. The course was designed and administered through UCLA’s Common Collaboration and Learning Environment (CCLE) and conducted using the Zoom© video conferencing platform. Students were asked to attend the first two class sessions in person at their respective institutions. Still, they were allowed to avail themselves of the flexibility of joining remotely for other sessions. Sharing professional and personal experiences related to health economics and global health concerns was evident in the required class session activities students discussed situational constraints and enablers. With access to the Zoom video conference capabilities, students worked outside class time with their peers to develop final projects. Classes were conducted over 16 weeks, with one 2–3 hour session per week, totalling 40 contact hours.

Offering joint courses helped solidify the partnership between the institutions, encouraged a more meaningful two-way transfer of knowledge, and offered opportunities to develop a system of collaboration and communication between the faculties.

## Sustainability

Sustainability can be thought of in two terms; the sustainability of the project and the sustainability of the partnership. Since the start of the partnership, UPM CPH has already been able to leverage the experience working on this project and the capacity development by successfully accessing a funding opportunity from the Philippine Department of Health to expand the research to an additional province in Mindanao and one city in Metro Manila. The team hopes to conduct periodic assessments of Primary Care readiness and needs to enable up-to-date monitoring and evaluation of national and sub-national NCD strategies and inform the improvement of services for vulnerable or underserved populations. UPM CPH also plans to develop a publicly accessible health database to be housed at UPM, which will be a source of timely and comprehensive population health data for use by policymakers, health program managers, researchers, media members, and others. It will help the Department of Health (DOH) – national and regional – and other stakeholders, such as private healthcare providers, identify which types of local health centres need help in improving their readiness and capability for tackling health problems through the newly enhanced tools.

UPM CPH also aims to empower partner state universities in Luzon, Visayas, and Mindanao to undertake surveys using methods and principles shared by colleagues from UCLA CHPR. Tarlac State University, Cebu Normal University, and Western Mindanao State University have agreed to be collaborating universities and expressed a wish to develop research skills. The project also plans to train university students to be the data enumerators and to train faculty and staff across the entire research cycle – from development to data collection to analysis to dissemination. The data collected is also designated as being available to interested UPM masters and doctoral students for their theses and dissertations. This partnership is seen as an investment in conducting more frequent population health surveillance of NCDs linked to primary care. Another critical sustainability piece of this project is the Advisory Board. The members of this Advisory Board have been crucial in providing feedback on local level implementation and advocating the team’s work to policy decision makers and other important stakeholders. Their involvement is vital to the utilisation of the project results and collaborative relationships fostering implementation, dissemination and future funding support.

As for UCLA CHPR and UPM CPH’s relationship after the project, the team is continually meeting monthly even after the project ended in June 2022 and has been collaborating on writing outcome papers. The faculties at UPM CPH are developing additional courses in systems thinking and implementation research. They are involving various members of this study as subject matter experts for the development of these courses as well as to provide case examples to be shared in the courses.

## Challenges

One of the main problems was that a global partnership required multiple layers of accountability and documentation to maintain the grant funding requirements. This sometimes delayed the procurement, limiting the team’s ability to move according to the project’s original timeline and fully achieve the capacity-building engagement activities envisioned originally. It was particularly challenging for UPM CPH because there was not a dedicated administrative personnel, and the project investigators and research team members who were part of the teaching faculty themselves had to provide time for those as well. The team overcame this challenge by hiring a research fellow, creating an advisory board, and hiring an administrative assistant to prepare documents, coordinate with finance and procurement sections of UPM. Seeking a team leader who was adaptable and accessible to the team members, the program required leadership change with the intention of keeping the study objectives on-track for completion. While these changes helped move the project forward, the overarching challenge of reconciling the achievement of research project objectives and simultaneously fulfiling the research project administration requirements remained a major hurdle.

The UCLA CHPR team faced similar challenges. The team is used to working on a fast-paced timeline for producing CHIS data and reports, but this project required much protracted decision-making processes. This was heightened by the COVID-19 pandemic where the team could not travel to the Philippines, curtailing in-person engagement and capacity-building activities originally planned. The survey was delayed for more than a year because of the shift of priority of the group towards pandemic response work in their institutions. This also created additional uncertainty on funding extension. To ensure the project did not derail due to the inability to carry out data collection efforts in the field, the team increased their meeting frequency and focused on changing data collection modality from in-person to online surveys for the health personnel. The population-level data collection was still on hold for a long time because of its in-person data collection modality. Despite these challenges, the project successfully completed two out of three original research aims during the study period and obtained a no-cost extension for the study aim related to population-level data collection.

## Discussion

There are notable examples of regional and global partnerships for capacity building and research training [8,11,20,21] such as the Consortium for Advanced Research Training in Africa (CARTA) [22–24] which has brought partnerships with multiple African universities and research institutions to strengthen research infrastructure and capacity in population and public health. The UCLA CHPR and UPM CPH partnership was unique in many ways and provides an example for the future direction of academic partnerships between HIC and LMIC institutions. The *first* is its unique opportunity to balance the traditional power asymmetry favouring HIC institutions due to the funding flow [2] by having the LMIC institution provide funding and research and study priorities, in this case from PCARI, an initiative of the Philippines government. With a greater recognition of the importance of the need to balance power in HIC-LMIC partnerships, we may expect more LMIC-initiated and funded collaborations in the future from different countries. The *second* is the setting of the research agenda by the LMIC institution themselves. This research collaboration came out of UPM CHP’s interest in developing their own research capacity on survey methodology around primary healthcare. This is a shift from the most commonly criticised aspect of the partnerships where the research agenda setting is usually led by funding calls from HIC, or by principal investigators in HIC institutions [2]. The *third* is team formation. Team members from UCLA CHPR had skill sets conducive to knowledge transfer while also bringing cultural awareness to the project. The *fourth* is co-teaching activities with the use of technology. The team offered hybrid classrooms simultaneously in both Los Angeles and Manila even before the pandemic. We anticipate more institutions implementing this approach, especially after the comfort of virtual and hybrid classrooms during the pandemic. We have already seen this shift with global health conferences allowing easier participation from LMICs [25,26].

It is understandable that often LMICs do not have such strong institutions which promote and fund international collaborations. Similarly, all partner universities may not have highly trained research faculties who have experience carrying out international research collaborations. Thus, reliance on HIC institutional partners for funding or initial project setup is likely not going to cease anytime soon. However, with new grant opportunities available through various organisations, including the Fogarty International Center in the National Institutes of Health [27], US academic institutions now have more opportunities to support research capacity building. Many capacity-building efforts are straightforward, such as sharing library resources, translating research papers into a local language, offering free education to students from LMIC partners, supporting research software including internet access, bi-directional flow of students, research agenda setting based on local needs, and opportunities in writing research articles and fair authorship [11,28,29], but these solutions still maintain the giver-receiver relationship and do not solve the power imbalance. The partnership should also strive towards building capacity for identifying and securing grants, executing research plans, managing software and library resources on its own, having secure data storage infrastructure, and being a training institution for researchers in their own country.

## Conclusion

Our description of the evolution of the academic partnership and the elements that have built a stronger connection between partnering institutions can be used as a model depicting new directionality and opportunities for other LMIC-HIC academic partnerships. We anticipate a growing number of LMIC-HIC academic collaborations use fair, equitable, and beneficial partnership models, incorporate capacity building as a core component, and use technology platforms to support collaborative work.

## Paper context

This paper describes a collaborative study designed and introduced by academic institutions from the Philippines and the US. The partnership was designed to build capacity for primary healthcare research and produce data for policy action for the prevention and care of non-communicable diseases (NCDs) in the Philippines. We describe the evolution of the academic partnership, implementation of the partnership project, and examine the elements that have built a stronger connection between the partnering institutions, along with the challenges.
